# Characteristics and posttransplant outcomes of patients with congenital and acquired von Willebrand disease and hemophilia A and with renal transplants

**DOI:** 10.1016/j.rpth.2025.103279

**Published:** 2025-12-02

**Authors:** Isabela Wen-Chi Chang, Mia Truman, Claire Yee, Leslie Padrnos

**Affiliations:** 1 Mayo Clinic Department of Internal Medicine, Scottsdale, Arizona, USA; 2 Mayo Clinic Department of Quantitative Health Sciences, Scottsdale, Arizona, USA; 3 Mayo Clinic Department of Hematology and Oncology, Scottsdale, Arizona, USA

**Keywords:** bleeding diathesis, factor VIII inhibitor, hemophilia A, renal transplantation, von Willebrand disease (VWD)

## Abstract

**Background:**

Bleeding disorders such as hemophilia and von Willebrand disease (VWD) have historically been associated with significant morbidity due to hemarthrosis, surgical bleeding, and transfusion requirements. With advances in hemostatic therapy, surgical outcomes have improved; however, data on renal transplantation in this population remain limited.

**Objectives:**

To assess clinical characteristics and adverse events in renal transplant patients with bleeding diathesis, focusing on primary endpoints: post-transplant bleeding, thrombotic events, and mortality. Secondary endpoints include readmissions, OR takebacks, and renal transplant rejections, including acute cellular rejection and antibody-mediated rejection.

**Methods:**

A retrospective chart review of renal transplant patients with bleeding diathesis across Mayo Clinic.

**Results:**

The cohort included 11 patients: hemophilia A (3/11) and VWD (8/11), with a mean Kidney Donor Profile Index of 37. Among patients with hemophilia A, 2 had congenital disease, and 1 had previously developed a factor VIII inhibitor that had resolved prior to transplantation. Among VWD patients, 6 had acquired VWD and 2 had type 1 VWD. No patients experienced arterial or venous thrombosis within 1 year. Beyond 1 year, 5 thrombotic events occurred in 4 patients (36.4%): 2 myocardial infarctions, 1 ischemic stroke, and 2 deep vein thromboses; no pulmonary emboli occurred. In the immediate postoperative period (days 0-30), 5 patients (45.5%) had bleeding events—4 major (80%) and 1 minor (20%). No bleeding occurred between days 30 and 365. After 1 year, 4 patients (36.4%) had nonallograft-related bleeding, primarily gastrointestinal. Readmission rates were 36.4% (0-30 days), 27.3% (30-90 days), and 9.1% (90-365 days). Surgical reintervention was required in 18.2% of patients. Rejection occurred in 3 patients (27.3%): 2 with acute cellular rejection, 1 with chronic cellular rejection, and 1 with antibody-mediated rejection. Overall mortality was 45%.

**Conclusion:**

Kidney transplant recipients with hemophilia and VWD are at significant perioperative bleeding risk, particularly from perinephric hematomas and intraoperative hemorrhage. The risk decreases after 30 days, but long-term monitoring for both bleeding and thrombosis remains crucial. Rejection rates appear comparable with those of the general transplant population.

## Essentials

• Bleeding disorders make surgery and kidney transplant care more complex and higher risk.
• This study evaluated kidney transplant outcomes in patients with hemophilia or von Willebrand disease.
• Nearly half of patients had major bleeding in the first 30 days after transplant surgery.
• After 30 days, bleeding declined, but late risks for blood clots and rejection remained significant.

## 1 Introduction

Renal transplantation is the most frequently performed solid organ transplant, surpassing liver and heart transplantation. In 2022, 25,499 kidney transplants were performed in the United States [1]. This number continues to rise due to the increasing prevalence of end-stage renal disease (ESRD), which currently affects approximately 808,000 individuals. Approximately 69% of these patients are maintained on long-term dialysis [1]. The leading causes of ESRD include long-standing diabetes mellitus and hypertension, as well as intrinsic renal conditions such as glomerulonephritis, focal segmental glomerulosclerosis, and obstructive uropathy [2].

Hemophilia A and B are rare X-linked genetic bleeding disorders caused by deficiencies in factor (F)VIII and IX, respectively, leading to spontaneous hemorrhage or bleeding with surgery or trauma [3,4]. Patients often experience severe functional consequences, including arthropathy from recurrent hemarthrosis. Advances in novel therapeutic strategies have significantly improved survival and quality of life for individuals with bleeding disorders. Further, von Willebrand Disease (VWD), another bleeding diathesis, results from a quantitative or qualitative deficiency in von Willebrand factor (VWF), a critical component of primary hemostasis. Defects in VWF lead to excessive bleeding, bruising, and prolonged hemorrhage following injury or surgery [5,6].

Historically, patients with hemophilia had reduced life expectancy, not only due to bleeding complications but also from transfusion-associated infections such as HIV and hepatitis C. Survival has since improved dramatically. For example, in an Italian study, the mortality rate ratio for hemophilia patients dropped from 1.98 in 1990-1999 to 1.08 in 2000-2007 [3]. Life expectancy also increased, reaching 71.2 years from a prior average of 64.0 years in the late 1990s [3]. As these patients live longer, they are increasingly affected by age-related conditions such as diabetes, hypertension, cancer, and renal disease. Nevertheless, large-scale studies evaluating renal disease progression and transplant outcomes in this population are lacking.

Some studies, such as Small et al. [7], suggest that most hemophilia patients do not develop renal failure requiring dialysis. However, as life expectancy rises and more high-risk individuals are considered for dialysis and transplantation, the number of bleeding disorder patients undergoing renal transplant is expected to increase [7,8]. Case reports have demonstrated successful renal transplantation in patients with hemophilia and VWD, with bleeding risk being the primary concern, especially in the postoperative setting [9]. Current perioperative guidelines recommend maintaining factor levels at 80% to 100% preoperatively, >50% during the first postoperative week, and >30% in the later postoperative period. As therapeutic advances continue to improve outcomes for patients with bleeding disorders, it is increasingly important to understand the unique challenges and outcomes associated with renal transplantation in this population.

## 2 Methods

A retrospective cohort review was conducted across the Mayo Clinic enterprise, including Mayo Clinic sites in Arizona, Minnesota, Florida, and the Mayo Clinic Health System. The study included patients with bleeding diatheses—specifically hemophilia A, hemophilia B, and VWD—who underwent renal transplantation between 2003 and 2024. A total of 11 patients, encompassing 12 transplant episodes, met the inclusion criteria. Of these, 3 patients were diagnosed with hemophilia A, and 8 patients had VWD. Among the patients with hemophilia A, 2 had congenital disease and 1 had previously developed a FVIII inhibitor that had resolved prior to transplantation. Among patients with VWD, 6 had acquired VWD and 2 had type 1 VWD.

The study used retrospectively collected data supplemented by a comprehensive review of clinical notes. Data collection included the etiology of ESRD, patient comorbidities, and adverse outcomes such as mortality, hospital readmissions, return to the operating room, and posttransplant thrombotic events (arterial and venous). Posttransplant bleeding events were assessed and graded according to International Society on Thrombosis and Haemostasis (ISTH) guidelines. Hemophilia A and B were graded for severity using World Federation of Hemophilia definitions: severe, <1%; moderate, 1%-5%; and mild, 5%-40% of normal pooled plasma factor levels [3]. The severity of VWD was graded by plasma VWF activity levels. Severe disease was defined as VWF activity of <5% of normal. Moderate disease was defined as 5% to 30% and mild disease as 30% to 50%. Patients with VWF activity of >50% were considered within the normal range. VWF activity was measured using ristocetin cofactor activity (or equivalent functional assay), with confirmatory testing by VWF antigen levels and FVIII activity where indicated. The severity of VWD was graded according to ristocetin cofactor activity (VWF:RCo). Severe VWD was defined as VWF:RCo of <5% of normal. Moderate VWD was defined as VWF:RCo of 5% to 30%, and mild VWD, as 30% to 50%. Patients with VWF:RCo >50% were considered within the normal range. VWF antigen (VWF:Ag) and FVIII activity levels were also measured to confirm diagnosis and assist in subtype classification. The FVIII studies and VWD testing were annotated in Table 2 if laboratories were done internally. Acquired vWD was diagnosed per ISTH criteria, which require new-onset bleeding, absence of a family history, abnormal vWF activity/antigen ratio (<0.7), and an associated condition.

**Table 2:** Preoperative management and coagulation studies.

Patient No.	Bleeding diathesis	Perioperative management	Fibrinogen (mg/dL)	PT/INR (s)	APTT (s)	Factor (F)VIII studies (prior to transplant)	VWD factor
1	VWD disease (acquired)	Humate	430	15.0/1.4	30	NA	NA^a^
2	Hemophilia A	Advate	288	13.3/1.0	26.7	FVIII, 48% (21% at nadir)^b^	NA
3	VWD disease (acquired)	None	444	13.6/1.2	36	FVIII, 98%	VWF Ag, 75% VWF activity, 54% Ristocetin cofactor, 48% (nadir 20%)
4	VWD disease (acquired)	FFP vs cryoprecipitate, Coagadex, or Humate	235	11.8/1.1	35	FVIII, 129%	VWF Ag, 189% VWF activity, 87% (nadir 47%) Ristocetin (nadir 28%)
5	VWD disease (type 1)	Humate	453	1.0	34	FVIII 52% (nadir at 31%)	VWF Ag, 49% (nadir 38%) VWF activity, 42% (nadir 25%) Ristocetin, 38% (nadir 23%)
6	Hemophilia A	FVIII (Helixate)	NA	13.0/0.99	40.6	FVIII, 55% (nadir 9%)	NA
7	Hemophilia A (FVIII inhibitor)—resolved	None	692	12.1/1.1	33	FVIII, 225% (nadir 28%) FVIII inhibitor (in Bethesda units), 8 (peak at 55)	NA
8	VWD disease (acquired)	None	279	17.3/1.5	33	FVIII, 183%	VWF Ag, 176% VWF activity, 112%^c^
9	VWD disease (acquired)	None	NA	15.4/1.2	32.2	FVIII, 110%	VWF Ag, 115% VWF activity, NA Ristocetin, 106%^c^
10	VWD disease (type 1)	Humate	NA	NA	NA	FVIII, 70%	VWF Ag, 53% Ristocetin, 46%^d^
11	VWD disease (acquired)	None	NA	11.5/1.0	23	FVIII, 350%	VWF Ag, 320% VWF activity, 211%^c^

Diagnosis was made externally.

FFP, fresh frozen plasma; NA, none available; VWD, von Willebrand disease; VWF, von Willebrand factor.

a This patient had mild hemophilia A (baseline FVIII ∼20%). Subsequently, transfusion-acquired hepatitis C led to cirrhosis and secondary coagulopathy. Precirrhosis factor levels are not available in our EMR, besides the baseline FVIII level.

b Plasma VWF multimer analysis reveals absence or decreased abundance of the highest molecular weight multimers (HMWMs), but no definitely increased abundance of lower-molecular-weight VWF multimers, suggesting an acquired rather than congenital abnormality of VWF multimers.

c Diagnosis was made externally. Repeat laboratories were not confirmed internally. The temporal relationship between Humate-P administration and VWF testing could not definitely be confirmed, and no preinfusion nadir was available.

Additional data included renal biopsy results, with Banff scores recorded for preimplantation (procurement), time-zero, 4-month, 1-year, and any additional biopsies. Rejection episodes were categorized as borderline acute cellular rejection (ACR), chronic cellular rejection, antibody-mediated rejection (AMR), chronic AMR, or mixed rejection.

Renal function was monitored through serum creatinine levels at 4 months, 1 year, 2 years, and 3 years posttransplant, along with the most recent available creatinine value. Graft loss and date of loss, if applicable, were also recorded. This study was approved by the institutional review board (IRB, 24-010112).

## 3 Results

### 3.1 Demographics

Of the 11 patients, 4 (36.4%) were females, and 7 (63.6%) were males. The majority were White (9/11, 81.8%), while 1 patient (9.1%) identified as Black or African American, and 1 (9.1%) as other. The mean age at the time of transplantation was 59 years (Table 1). Regarding smoking history, 9 patients (81.8%) were former-smokers, and 2 (18.2%) were never-smokers.

**Table 1:** Prerenal transplant clinical details.

Patient No.	Age (y) and sex	Race	Ethnicity	Bleeding diathesis	Other concomitant bleeding	Severity	ESRD reason	Renal transplant waitlist time (d)	Dialysis prior; hemodialysis vs peritoneal dialysis	Length of dialysis prior to transplant
1	42 M	White	Non-Hispanic or Latino	VWD disease	None	Unspecified; acquired	FSGS	10	HD	7846
2	52 M	Other	Hispanic or Latino	Hemophilia A	None	Mild	Diabetes	8	HD	20
3	60 F	White	Non-Hispanic or Latino	VWD disease	Essential thrombocytosis (JAK2 negative)	Mild; acquired	Amyloidosis	435	HD	1069
4	55 M	White	Non-Hispanic or Latino	VWD disease	Factor X deficiency	Mild; acquired	Amyloidosis	60	PD	691
5	62 M	White	Non-Hispanic or Latino	VWD disease	None	Mild; type 1	Membranous nephropathy	0	None	966
6	74 M	White	Non-Hispanic or Latino	Hemophilia A	None	Mild; acquired	Diabetes and hypertension	636	HD	344
7	72 M	White	Non-Hispanic or Latino	Hemophilia A	None	Mild; acquired	Membranous nephropathy; possible overlying IgA nephropathy	587	HD	901
8	68 F	Black or African American	Non-Hispanic or Latin American	VWD disease	None	Mild; acquired	Diabetes and HTN	342	HD	2535
9	48 F	White	Non-Hispanic or Latin American	VWD disease	None	Mild; acquired	Diabetes	368	HD	2299
10	59 F	White	Non-Hispanic or Latin American	VWD disease	None	Mild; type 1	Cyclosporine toxicity	NA	NA	0
11	57 M	White	Non-Hispanic or Latin American	VWD disease	None	Mild; acquired	MPGN	266	NA	0

FSGS, focal segmental glomerulosclerosis; HD, hemodialysis; HTN, hypertension; MPGN, membranous proliferative glomerulonephritis; NA, not available; PD, peritoneal dialysis; VWD, von Willebrand disorder.

With respect to bleeding disorders, 3 patients (27.3%) had hemophilia A, of which 2 were congenital and 1 had previously developed a FVIII inhibitor that had resolved before transplantation. No patients had hemophilia B. Eight patients (72.7%) had VWD—6 with acquired VWD and 2 with type 1 VWD. Three patients (patients 8, 9, and 11) (Table 2) were initially categorized as having acquired VWD; however, retrospective review indicated that their laboratory findings may represent assay variability, such as in stress states or in acute phase responses, may represent assay variability rather than true acquired VWD.

Common comorbidities included hypertension (9/11, 81.8%) and anemia (11/11, 100%). Thrombocytopenia was present in 4 patients (36.4%), and thrombocytosis was notable in 3 of 11 patients (27%). Liver disease was documented in 3 patients (27.3%), all with hepatitis C; 2 developed cirrhosis, including 1 who required liver transplantation. Three patients (27.3%) had diabetes mellitus, and 3 (27.3%) had a history of cancer, although none required chemotherapy or radiation. Two patients (18.2%) had amyloidosis.

Anticoagulation use was limited. One patient was on warfarin for a mechanical aortic valve, transitioned to heparin around the time of transplant. Two others had prior anticoagulant exposure (apixaban and warfarin) but were discontinued months before transplant due to bleeding complications or completion of treatment.

The median time on the transplant waitlist was 355 days (IQR, 102-549 days; Table 1. The median dialysis duration before transplantation was 691 days (IQR, 10-1684 days; Table 1). Six patients (54.5%) received living donor transplants, and 5 (45.5%) received deceased donor transplants.

### 3.2 Transplant details

One patient (9.1%) underwent a simultaneous liver-kidney transplant, while 10 received kidney-only transplants (Table 3). Five patients (45.5%) had a history of prior renal transplantation, and 2 (18.2%) had prior nonrenal organ transplants (1 liver, 1 lung). One kidney came from an acute kidney injury donor, and 2 from hepatitis C–positive donors (9.1% and 18.2%, respectively) (Table 4). The average Kidney Donor Profile Index was 37, and the mean HLA mismatch was 2.75 of 6 (Table 4).

**Table 3:** Operative details.

Patient No.	Prior transplant course	Transplant and type (DCD vs alive)	Redo transplant	AKI donor	Hepatitis C–positive donor	HLA mismatch	KDPI	Estimated blood loss (mL)	Hospitalization length (d)	Blood products given
1	RT × 2	DCD RT	Yes	No	Yes	NA	50	1200	17	10 units of pRBC 3 units of PLTs
2	None	DCD RT; SLK	No	No	No	NA	18	NA	33	23 units of pRBC 4 units of FFP 7 units of PLTs 20 units of cryoprecipitate
3	None	Live RT	No	No	No	NA	NA	NA	3	None
4	None	Live RT	No	No	No	2/6	NA	150	6	8 units of pRBCs 4 units of FFP 1 unit of PLTs
5	None	Live RT	No	No	No	6/6	NA	100	9	2 units of pRBCs
6	None	DCD RT	No	Yes	No	NA	69	NA	5	None
7	None	Live RT	No	No	No	NA	NA	75	3	None
8	Prior LT and RT	DCD RT	Yes	No	Yes	NA	29	400	5	2 units of pRBCs
9	Prior RT	DCD RT	Yes	No	No	NA	19	NA	8	7 units of pRBCs
10	Prior lung transplant	Live RT	No	No	No	0/6	NA	NA	7	2 units of pRBCs; 2 units of FFP
11	Prior RT × 2	Live RT	Yes	No	No	3/6	NA	NA	6	None

AKI, acute kidney injury; DCD, donation after circulatory death; FFP, fresh frozen plasma; HLA, human leukocyte antigen; KDPI, Kidney Donor Profile Index; LT, liver transplant; NA, none available; pRBC, packed red blood cell; PLT, platelet; RT, renal transplant.

**Table 4:** Transplant information.

Characteristic	*N* = 11^a^
Transplant date	2003-2024
Transplant type	
Live donor (6/6 solitary renal transplant)	6 (55)
Deceased donor (4/5 solitary renal transplant 1/5 simultaneous liver-kidney transplant)	5 (45)
Prior transplant history: renal transplant	5 (45)
Prior transplant history: nonrenal transplant	2 (18)
Redo transplant	5 (45)
AKI donor	1 (9.1)
Hepatitis C virus–infected donor	2 (18)
KDPI (average)	37
HLA mismatch	
0/6	1 (9.1)
2/6	1 (9.1)
3/6	1 (9.1)
6/6	1 (9.1)
Unknown	7 (63)
Thrombosis (0-30 d)	
None	11 (100)
Thrombosis (30-90 d)	
None	11 (100)
Thrombosis (90-365)	
None	11 (100)
Thrombosis (>365 d)	
DVT (venous)	2 (20)
PE (venous)	0 (0)
MI (arterial)	2 (20)
Stroke (arterial)	1 (10)
Other	0 (0)
None	6 (60)
Unknown	1 (10)
OR (0-30 d)	
None	9 (82)
Take backs related to allograft	1 (9.1)
Other surgeries	1 (9.1)
OR (30-90 d)	
None	10 (91)
Take backs related to allograft	0 (0)
Other surgeries	1 (9.1)
OR (90-365 d)	
None	11 (100)
Take backs related to allograft	0 (0)
Other surgeries	0 (0)
Readmissions (0-30 d)	4 (36)
Readmissions (30-90 d)	3 (27)
Bleeding (0-30 d)	
No	6 (55)
Yes: related to allograft	5 (45)
Bleeding (0-30 d) character	
Major	4 (80)
Minor B (clinically not relevant)	1 (20)
Bleeding (30-90 d)	
No	11 (100)
Bleeding (30-90 d) character	
No	11 (100)
Bleeding (90-365 d)	
No	11 (100)
Bleeding (>365 d)	
No	6 (55)
Yes: not related to allograft	4 (36)
Unknown	1 (9)
Bleeding (>365 d) character	
Major	4 (100)
Limb ischemia	1 (9.1)
Immunosuppressive therapy at induction	
Alemtuzumab	1 (9.1)
Thymoglobulin	4 (36)
Basliximab	6 (55)
Immunosuppression: Prograf	11 (100)
Immunosuppression: Cellcept	11 (100)
Immunosuppression: prednisone	11 (100)
Immunosuppression: everolimus	0 (0)
Immunosuppression: serolimus	0 (0)
Immunosuppression: belatacept	0 (0)
Immunosuppression: cyclosporine	0 (0)
Immunosuppression: other	0 (0)
Graft failure	5 (45)
Delayed graft function	1 (9.1)
Graft failure	
Acute	2 (100)
Unknown	9
Cause of graft failure	
Other	5 (100)
Unknown	6
Prolonged use of antimicrobials	2 (18)
Use of TPO	0 (0)
Use of GCSF	2 (18)
Use of ESA	5 (45)
Death	5 (45)
Rejection	2 (29)
Unknown	4

AKI, acute kidney injury; DVT, deep vein thrombosis; ESA, erythropoietin-stimulating agent; GCSF, granulocyte colony-stimulating factor; HLA, human leukocyte antigen; KDPI, Kidney Donor Profile Index; MI, myocardial infarction; OR, operating room; PE, pulmonary embolism; TPO, thrombopoietin.

a Range; *n* (%).

### 3.3 Perioperative and postoperative management

Patients with hemophilia A received FVIII replacement perioperatively (Advate or Helixate) with close monitoring. Perioperative management for patients with VWD was less standardized.

Perioperative hemostatic management was directed by the hematology service for all patients with congenital or acquired bleeding disorders. Supplementation protocols and monitoring data were reviewed in detail to assess adequacy of replacement and to contextualize the observed bleeding outcomes. In patients with congenital disorders, perioperative supplementation was consistently implemented with the goal of maintaining hemostatic factor levels at or approximately 100% of normal. For example, patient 2, who had hemophilia A, received recombinant FVIII (Advate) replacement therapy with a target activity level of 100% prior to surgery. Factor VIII levels were monitored daily, and the dosing regimen was escalated from intermittent bolus administration to continuous infusion to sustain therapeutic levels throughout the perioperative period. Infusion rates were titrated based on laboratory monitoring to ensure optimal replacement, while minimizing the risk of volume overload. Similarly, patient 6, with hemophilia A, underwent preprocedural factor testing to confirm appropriate dosing, and dosing regimen was titrated to FVIII activity levels at 100%.

Patient 5, who had type 1 VWD, received Humate-P as a bolus 10 to 15 minutes before surgical incision, targeting VWF and FVIII activity at 100% of normal. Laboratory testing was performed 5 minutes, 4 hours, and 24 hours after the initial bolus, and daily thereafter for 4 to 5 postoperative days, allowing real-time dosing adjustments based on VWF and FVIII activity levels. Replacement therapy was guided carefully to balance bleeding prevention and avoidance of supratherapeutic correction that could predispose thrombosis.

Patient 10, who also had type 1 VWD, required perioperative correction of RCo activity from 50% to approximately 100%. Based on standard pharmacokinetic calculations, she received approximately 2000 units of Humate-P intravenously prior to surgery, followed by every-12-hour dosing with serial monitoring of VWF antigen, RCo, and FVIII activity to maintain target levels throughout the perioperative period.

Mean estimated blood loss was 385 mL (documented in 5 of 11 cases). Patients received an average of 4.9 units of packed red blood cells, 1 unit of platelets, and 0.9 units of fresh frozen plasma. The mean hospital stay was 9.3 days (6.9 days excluding the simultaneous liver-kidney transplant) (Table 3).

For induction immunosuppression, 6 patients (55%) received basiliximab, 4 (36%) thymoglobulin, and 1 (9%) alemtuzumab (Table 4). At discharge, all were maintained on tacrolimus, mycophenolate mofetil, and prednisone (Table 4).

### 3.4 Posttransplant outcomes

There were no thrombotic events in the immediate 30-day postoperative period. However, 5 late events (>1 year) occurred: 2 myocardial infarctions, 1 ischemic stroke, and 2 deep vein thromboses (36%) (Table 4).

Bleeding complications occurred in 5 patients (45%) within 30 days. Four were major and 1 minor (per ISTH criteria). Complications included perinephric hematomas and soft tissue hemorrhage; 1 patient with simultaneous liver-kidney transplant developed massive intraoperative bleeding requiring >20 units of packed red blood cells. No bleeding occurred between days 30 and 365. Four patients (36%) experienced late bleeding (>1 year), primarily gastrointestinal from arteriovenous malformations (Table 3).

Two patients (18%) required operating room (OR) takeback for allograft-related or other surgical issues. One patient (9%) required OR takeback related to the allograft with reimplanation of transplant ureter and renal biopsy and another (9%) underwent OR takeback unrelated to the allograft. No takebacks occurred between day 90 and 365 postoperatively for allograft-related issues (Table 4).

Hospital readmissions occurred in 4 of 11 patients (36%) between postoperative days 0 and 30, and in 3 patients (27%) between days 30 and 90. Hospital readmissions included acute kidney injury, hyperkalemia, and infections, including BK nephropathy (Table 4).

Rejection occurred in 3 patients (27%): 2 acute cellular, 1 chronic cellular, and 1 AMR. No cases of chronic AMR or mixed rejection were seen (Table 5).

**Table 5:** Biopsy information.

Characteristic	*N* = 11^a^
ACR	2/11
Chronic cellular rejection	1/11
AMR	1/11
Chronic AMR	0
Mixed ACR and AMR	0

ACR, acute cellular rejection; AMR, antibody-mediated rejection.

a *n* (%); range.

During follow-up, 5 of 11 patients (45%) died: 2 from unclear causes, 1 from disseminated nocardiosis, 1 from pulmonary edema, and 1 from cardiac arrest (Table 4).

## 4 Discussion

There have been few documented case reports of patients with bleeding diathesis undergoing renal transplantation. One case describes a Western Indian patient with hemophilia A who successfully underwent a live unrelated renal transplantation with FVIII replacement from preoperative day 1 to postoperative day 12 [10]. Another report detailed a Moroccan male with hemophilia A who also had a successful renal transplantation with stable graft function and no clinical complications [11]. Patients with VWD have likewise undergone successful renal transplantation [12]. However, the literature remains limited to case reports, with no large cohort studies systematically investigating renal transplantation in this population.

Patients with bleeding diathesis face a heightened perioperative risk, particularly from perinephric hematomas and hemorrhage. The greatest vulnerability occurs within the first 30 days postoperatively. In our cohort, 45% experienced acute bleeding, most commonly perinephric hematomas. One patient developed an extensive hemorrhage involving the abdomen and thighs. Beyond 1 year, 80% of those with prior bleeding had major bleeding episodes, and by day 365, 40% (4/10) developed nonallograft-related bleeding, primarily gastrointestinal, while 60% remained free of bleeding events. These findings underscore that although risk diminishes after the immediate postoperative period, long-term vigilance remains essential. In the literature, postoperative hemorrage account for approximately 14% of the patients who undergo renal transplant; with our cohort, that risk is tripled that of the patients in the immediate postoperative timeline [13]. A recent retrospective cohort study evaluated major bleeding events in renal transplant patients—excluding those with congenital bleeding disorders, multiorgan transplants, or prior renal transplants—and found that 38.1% of anticoagulated patients experienced major bleeding compared with 25.7% of nonanticoagulated patients [14].

Importantly, no thrombotic events occurred during the early postoperative period. However, 4 thrombotic events (36%) were observed remotely, more than 1 year after transplantation, including myocardial infarction (18%), stroke (9%), and deep vein thrombosis (9%). This distinction highlights that the perioperative period is dominated by bleeding risk, whereas longer-term follow-up reveals a shift toward thrombotic complications. These outcomes mirror observations in nonbleeding disorder transplant populations, where cardiovascular disease remains a major late complication.

With respect to secondary endpoints, 36% of patients required readmission within 0 and 30 days, 18% between 30 and 90 days, and 9.1% between 90 and 365 days. Regarding graft outcomes, 18.2% experienced ACR, 9.1% chronic ACR, and 9.1% AMR. These rates are consistent with published data. For example, a Bogotá, Colombia, cohort reported an ACR incidence of 14.3% [15]. National data from Organ Procurement and Transplantation Network/United Network for Organ Sharing similarly cite ACR rates of 12% to 18% among live donor recipients and 14% to 30% among deceased donor recipients, particularly within 6 months of transplantation. Our findings, although limited by sample size, are thus aligned with global experience.

### 4.1 Limitations and future directions

The most significant limitation of our study is the small cohort size, which substantially limits statistical power and generalizability. With only 11 patients over 21 years, the ability to detect meaningful associations is restricted, and results may be disproportionately influenced by outliers. Additionally, heterogeneity in the patient population—combining congenital hemophilia A with acquired and type 1 VWD—introduces biological and clinical variability that weakens internal validity, as outcomes and risk profiles are likely distinct across subgroups. In addition, 3 patients (patients 8, 9, and 11) (Table 2) underscore the diagnostic ambiguity inherent to acquired VWD, particularly when classification relies solely on VWF Ag/activity ratio in the absence of clinical bleeding phenotypes. In these settings supraphysiologic VWF and FVIII levels, it may represent overdiagnosis or laboratory artifact or in scenarios such a stress response. The single-center retrospective design, while common in rare disease research, raises concerns regarding selection bias, practice-specific variability, and nonstandardized data collection. Furthermore, some diagnostic uncertainties in patient classification and discrepancies in reported factor deficiencies highlight the need for standardized diagnostic criteria in this setting.

Our findings suggest that indiscriminate screening for occult bleeding disorders before renal transplantation is not warranted; rather, a focused approach based on bleeding history, laboratory abnormalities, or associated conditions is preferable. Overdiagnosis, particularly of acquired VWD, may expose patients to unnecessary supplementation and thrombotic risk. Standardization of pretransplant coagulation testing and interpretation, including uniform use of VWF antigen/activity assays and confirmatory testing, is essential to balance safety and accuracy.

Despite these limitations, the study provides valuable preliminary insights into the feasibility of renal transplantation in patients with bleeding disorders and underscores the critical need for tailored hemostatic protocols and long-term surveillance. Larger multicenter collaborations, including coordination with United Network for Organ Sharing, are essential to build a comprehensive registry and clarify both short- and long-term risks in this growing patient population. Prospective studies with standardized care protocols and matched controls will be necessary to validate and extend these findings.

## 5 Conclusion

Renal transplantation can be safely performed in patients with bleeding diathesis, with acceptable perioperative morbidity. Although bleeding is the dominant risk early after surgery, late thrombotic events emerge as clinically significant. Close multidisciplinary follow-up is therefore critical to optimize both patient and graft outcomes.
